# A population-based study on health-related quality of life among urban community residents in Shenyang, Northeast of China

**DOI:** 10.1186/s12889-015-2238-8

**Published:** 2015-09-19

**Authors:** Tian Song, Yan-wei Ding, Yan Sun, Yi-Ni He, Dian-jun Qi, Ying Wu, Bin Wu, Lang Lang, Kai Yu, Xin Zhao, Liang-liang Zhu, Shuang Wang, Xiao-Song Yu

**Affiliations:** General Medical Practice of the First Hospital of China Medical University, 155 North Nanjing Street, Heping District, Shenyang, Liaoning Province 110001 PR China

## Abstract

**Background:**

Due to the rising standard of living environment and advances in public health and medical care in China, it has been a tendency in recent years that health-related quality of life (HRQoL) has been increasingly acknowledged in community health management. However, large-scale population-based study on evaluating HQRoL in northeast of China was not conducted. This article aims to investigate the HRQoL in community residents in Northeast China and explore the associated factors.

**Methods:**

Stratified multiple-stage sampling method was used in the cross-sectional survey to investigate HRQoL of community residents in northeast of China. Univariate analysis and multiple linear regressions were used to analyze the factors associated to HRQoL of the community residents.

**Results:**

The results were confirmed that HRQoL in general population was well performed for the first time in northeast of China in a large scale population. Community residents had better mental health than physical health. The factors influencing HRQoL included gender, age, educational level, marital status, ethnic group, chronic disease status, having breakfast frequency weekly and sleep quality. However, drinking and smoking habits did not affect residents’ HRQoL.

**Conclusions:**

In this study, the result of the large-scale survey was satisfactory in northeast of China, providing HRQoL status of community residents. Policies on specific health management in community public health would emphasize on lifestyle behaviors especially eating habits in order to improving HRQoL.

## Background

It has been a tendency in recent studies that health-related quality of life (HRQoL) has attracted more attentions because of the rising standard of living environment and advances in public health and medical care, which is an individuals’ satisfaction or happiness with the dimensions of life insofar as they affect or are affected by health [1]. Certain surveys and population-based studies were conducted in different regions of China [2–9]. Individuals’ physical, emotional and social functioning has been evaluated in different degree of HRQoL in China [10]. High reliability and validity was confirmed in studies and measured by SF-36 in Chinese HRQoL studies. HRQoL has also been measured for health management and clinical advices of chronic diseases [11–14]. Elderly adults’ HRQoL were focused on different conditions for the reason that the aging group has expanded tremendously in decades worldwide especially in China, the largest population scale worldwide [15–19].

Northeast area was an important industrial base in China. It has been making a significant contribution in economic development and social construction since the 1950s. However, great changes have taken place in humanities and natural environment because social structure has been greatly changed since the 1990s. The living environment, income status and lifestyle of residents changed a lot. Thus, the quality of life and health has changed. The prevalence of chronic diseases rate of urban residents in Liaoning province has dramatically increased in recent decades (from 27.49 to 41.86 %), much higher than the country’s results (28.3 %) in China’s fourth national health services survey in 2008 [20, 21]. Investigations of HRQoL in northeast of China could provide specific public health policy in northeast area in order to improve individuals’ health. In addition, factors associated with HRQoL could be applied in clinical practice when health management is occupying. There have been studies used successfully in specific groups such as college teachers, caregivers and poverty individuals in northeast China in recent years [22–24]. However, study in general urban population has not been specifically conducted yet. Thus, this article is aimed at assessing the health-related quality of life in the population of urban community residents in Shenyang, northeast of China, and evaluating the related factors influenced by HRQoL.

## Methods

### Study sample

This study was the first study evaluating health-related quality of life in large scale population-based survey in northeast of China. From July 2013 to March 2014, a cross-sectional survey was performed in Shenyang, Liaoning province, the largest city in northeast China. Being the social and economical core city in northeast China, Shenyang has a reputation as “the Oriental Ruhr”. It consists of five urban districts, five suburban districts and four rural districts. This survey was conducted in the five main urban residential areas in Shenyang, namely, Heping district, Shenhe district, Dadong district, Huanggu district and Tiexi district.

A randomized stratified multiple-stage sampling method was used in this survey, involving stratification sampling method followed by systematic sampling method. In the first stage, with respect to geographical scale and contribution in each district, cluster sampling was used to select representative community health centers which medical care coverage was more than a hundred thousand in population in each district. Furthermore, the average number of outpatients in those community health centers was more than 50. In the second stage, according to the sampling results, 27 community health centers were selected randomly in five districts, including seven centers in Heping district, four centers in Shenhe district, nine centers in Dadong district, six centers in Huanggu district and two centers in Tiexi district. Finally, participants were randomly selected from each community health center in their coverage of population. Residents who went to the community health centers were randomly selected to participate in the face-to-face investigation. A total of 6,000 residents, 1,200 from each district, were selected to participate in this survey. Residents over the age of 18 were allowed to participate in this survey. All participants are able to read or understand Chinese characters. 5,645 community residents were effectively responded to the survey. The respond rate was 94.08 %. 285 residents were declined because their questionnaires were uncompleted more than 50 % in each dimension of the questionnaire. 5,360 questionnaires were available.

All the participants who were well-informed about the content and aim of the study signed informed consent about the project. Trained project doctors or surveyors interviewed the participants face to face in order to complete the questionnaire. The procedures were approved by the ethical standards of the Committee in the First Hospital of Human Experimentation of China Medical University.

### Measurement

#### Measurements of HRQoL

The 36-item Short Form Health Survey (SF-36) has been developed and most commonly measured in the studies of HRQoL. SF-36 has been adapted and applied for health status and outcomes in different countries since it has been translated into different languages to be measured. The SF-36 (v2) Chinese version (from quality metric incorporated) was widely used in the studies of HRQoL. The reliability and validity had been proved to be acceptable in different surveys [10, 17, 25, 26]. It examines eight different dimensions of health: physical function (PF), role limitations due to physical problems (RP), bodily pain (BP), general health (GH), vitality (VT), social function (SF), role limitations due to emotional problems (RE) and mental health (MT). It was also divided into two dimensions: Physical Component Summary (PCS) and Mental Component Summary (MCS). Scores in the SF-36 range from 0 to 100, representing a better health state with higher scores. The PCS and MCS scores were calculated by the standard scoring algorithms of Hong Kong [10]. Missing values were calculated as follows: if the items were completed for 50 % or more in each dimension, the missing values were computed by the mean value to complete the missing items. In the other hand, the score was excluded from analysis in statistics if more than 50 % items were not completed in each dimension.

#### Measurements of socio-demographic and lifestyle behaviors

Socio-demographic characteristic and lifestyle were also variables to be evaluated. Socio-demographic characteristic included gender, age, marital status, educational level, ethnic group, living environment last year, and medical insurance. Age groups were divided into seven categories: 18 to 29, 30 to 39, 40 to 49, 50 to 59, 60 to 69, 70 to 79 and over 80 years. Marital status was divided into three categories: currently unmarried, currently married and divorced or widowed. Educational level, the highest level participants attained, was grouped into five categories. Respondents graduated from: primary school or lower, junior middle school, senior high school, college and university or higher. Ethnic group included Han, Hui, Miao, Wei, Man and other ethnic groups. Living environment last year included urban, suburban and rural areas. Medical insurance types were divided into four categories: the new rural cooperative medical care, medical insurance for urban residents and workers, expenses for one’s own and the others.

Lifestyle behavior included smoking, drinking, quality of sleep, frequency of having breakfast weekly and status on chronic disease. Smoking habits was divided into three categories: smoking, ex-smoking and non-smoking. Respondents who had smoked 1 cigarette or more per day for 1 year or more were seen as smokers. Drinking habit was grouped into drinking and non-drinking. Individuals who had drunk beer, liquor, yellow rice wine or red wine per week for more than 12 months were defined as drinking habits. Sleep quality in the last month has been evaluated into four statuses: very good, still good, not good and very poor. Frequency of having breakfast weekly means how often individuals have breakfast in one week for years. Less than 2 days, 2–3 days, 4–5 days and more than 5 days were divided to measure the frequency. Participants who have one or more chronic diseases diagnosed in the clinics were defined as in chronic disease status. Chronic disease included the most common chronic conditions, which were hypertension, diabetes, hyperlipidemia, cardiovascular and cerebrovascular diseases, pneumonia, asthma, chronic bronchitis, emphysema, pulmonary tuberculosis, lung cancer, bronchial asthma gallstones, cholecystitis and digestive ulcer.

### Statistical analysis

Epidata 3.01 was used to input and manage the data in the survey. SPSS 16.0 version for windows carried out statistical analysis that included socio-demographic characteristics and lifestyle behaviors and scores of SF-36. Frequencies (N), percentages (%), means and standard deviations (SD) were descriptive denoted for statistics. Cronbach’s coefficient values were used to measure internal reliabilities of HRQoL in our study. Independent *t*-test was used to estimate differences in gender, chronic disease status and drink group result. One-way ANOVA test was used to assess comparisons with respect to socio-demographic characteristic groups including age, educational level, marital status, living environment and medical insurance, and lifestyle behaviors groups including smoking, frequency of having breakfast weekly, and sleep quality with P value below 0.05 was considered significantly different in this study. Multiple linear regressions were chosen to analyze the relationship between HRQoL and lifestyle factors and socio-demographic characteristics, standard Beta values and *P* value for each dimension in the regression model were provided. *P* value below 0.05 was considered significantly contributions to the HRQoL. Gender (male or female), drinking habit (yes or no) and chronic disease (yes or no) were the binomial variables. Educational level was assessed with a set of four dummy variables reflecting college, senior high school, junior middle school and primary school or lower, with university or higher as the reference category; marital status was assessed with a set of two dummy variables reflecting currently married and divorced or widowed, with currently unmarried as the reference category; ethnic group was assessed with a set of five dummy variables reflecting Hui, Miao, Wei, Man and Zhuang, with Han as the reference category; medical insurance assessed a set of three dummy variables reflecting the new rural cooperative medical care, medical insurance for urban residents and workers, with expenses of one’s own as the reference category; having breakfast weekly assessed with a set of three dummy variables reflecting less than 2, 2–3 and 4–5 days, with more than 5 days as the reference category; sleep quality also assessed with a set of three dummy variables reflecting fairly good, not good and very poor, with very good as the reference category.

## Results

### Description of the socio-demographic characteristic and lifestyle behaviors

Socio-demographic and lifestyle behaviors variables of participants in this study were shown in Table 1. A total 5,360 individuals effectively completed the questionnaire in this survey, of whom 3,255 (42.1 %) were males and 3,105 (57.9 %) were females. The average age of the residents was 53.43 (±14.16) years old, ranging from 19 to 91 years. 84.3 % individuals were currently married, and 30.8 % received junior middle school education. 94.8 % were in Han ethnic group. In other ethnic groups, Man was with the most individuals(2.6 %) while “other ethnic groups” (1.5 %) contained mostly Chaoxian and Menggu. 91.3 % residents live in Shenyang urban district, and 86.4 % possess medical insurance for urban residents and workers.

**Table 1:** frequency distribution of socio-demographic characteristics and lifestyle behaviors in northeast China (*N* = 5,360)

Variables (groups)	Number	Percent	Variables	Number	Percent
Gender			Living environment		
Male	2555	42.1	Urban	4895	91.3
Female	3105	57.9	Suburban	338	6.3
Age (year)			Rural	127	2.4
18–29	347	6.5	Medical insurance		
30–39	640	11.9	Expenses of one’s own	383	7.1
40–49	899	16.8	The new rural cooperative medical care	210	3.9
50–59	1452	27.1	Medical insurance for urban residents and workers	4631	86.4
60–69	1321	24.6	Others	136	2.5
70–79	624	11.6	Chronic disease status		
80-	77	1.4	Yes	2760	51.5
Education level			No	2600	48.5
Elementary school or lower	335	6.2	Smoke		
Junior middle school	1653	30.8	Never	4688	87.5
Senior middle school	1405	26.2	Yes	557	10.4
College	114	20.8	Give up	115	2.1
University or higher	853	15.9	Drink		
Marital status			Yes	695	13.0
Not married yet	621	11.6	No	4665	87.0
Married currently	4516	84.3	Breakfast frequency weekly		
Divorced or widowed	223	4.2	Less than 2 days	4825	90.0
Ethnic group			2–3days	370	6.9
Han	5083	94.8	4–5days	98	1.8
Hui	42	0.8	More than 5 days	67	1.2
Miao	9	0.2	Sleep quality		
Wei	8	0.1	Very good	848	15.8
Man	138	2.6	Fairly good	4224	78.8
Others	80	1.5	Fairy bad	265	4.9
			Very bad	23	0.4

51.5 % participants suffered one chronic disease or more. 87.5 % were non-smoking, nearly the same as non-drinking individuals (87.0 %). Furthermore, 78.8 % residents have a good sleep at night. However, 90.0 % residents have breakfast less than 2 days weekly.

### Residents’ scores of HRQoL

The scores in different dimensions of SF-36 were shown in Table 2. In addition, the internal reliability of SF-36 ranged from 0.277 to 0.892, which was measured by Cronbach’s α. Comparisons among different dimensions of socio-demographic characters and lifestyle behaviors were found in Table 3: (1) the health-related quality of life was reduced with increasing age. In addition, females had better mental health than males, especially in RE and MH dimensions. (2) Residents who are currently unmarried had higher physical component summary (PCS) scores than residents in other marital status; residents who are currently married had highest scores in mental component summary (MCS); residents who got bachelor or higher degree had higher scores than others as well. (3) Residents who suffered one or more chronic diseases had higher scores in SF-36 dimensions.

**Table 2:** HRQOL scores and reliability of SF-36 dimensions of Northeast China (Shenyang) residents (*N* = 5360)

Scale	Means	SD	Cronbach’s α
PF	85.68	18.20	0.892
RP	84.51	20.64	0.865
BP	84.51	16.20	0.806
GH	68.26	19.19	0.752
VT	74.41	16.87	0.277
SF	94.35	20.82	0.332
RE	85.73	20.45	0.833
MH	73.99	17.88	0.705
PCS	**49.35**	**9.06**	
MCS	**55.25**	**8.91**	

*SD=* standard deviation, *PF=* physical functioning, *RP=* role limitations due to physical problems, *BP=* bodily pain, *GH=* general health, *VT=* vitality, *SF*= social functioning, *RE=* role limitations due to emotional problems, *MH=* mental health, *PCS=* physical component summary, *MCS=* mental component summary

The mean scores and standard deviation scores of the MCS and PCS are marked as bold words

**Table 3:** HRQOL scores in frequency distributions of socio-demographic characteristics and lifestyle behaviors in northeast China

Variables	PF	RP	BP	GH	VT	SF	RE	MH	PCS	MCS
	Means									
	(SD)									
	t/F									
	P									
Gender										
Male	**86.33**	**85.25**	**84.93**	**68.99**	**74.59**	**94.43**	85.69	71.17	**50.45**	49.93
	(17.66)	(19.30)	(16.23)	(19.16)	(16.8)	(20.91)	(20.41)	(17.87)	(7.77)	(9.02)
Female	85.21	83.97	84.20	67.73	74.28	94.29	**85.76**	**73.85**	49.69	**50.07**
	(18.58)	(21.54)	(16.16)	(19.21)	(16.87)	(20.76)	(20.48)	(17.89)	(8.14)	(9.02)
T	2.213*	2.27*	1.631	2.378*	0.657	0.245	−0.139	0.648	3.274	−0485
Age (year)										
18–29	**89.56**	**86.40**	84.95	68.80	73.68	94.13	**88.23**	73.50	51.20	46.63
	(16.61)	(13.05)	(16.29)	(17.78)	(16.38)	(21.40)	(11.05)	(17.84)	(6.79)	(8.22)
30–39	88.30	85.45	**86.85**	**70.10**	74.89	95.49	85.56	74.08	**51.51**	49.68
	(18.30)	(13.39)	(15.82)	(19.06)	(17.25)	(21.04)	(15.48)	(18.35)	(6.99)	(8.64)
40–49	86.58	86.03	84.93	70.07	74.02	94.42	85.14	74.12	50.83	49.63
	(18.17)	(18.00)	(15.63)	(19.83)	(17.64)	(20.64)	(20.53)	(17.62)	(7.78)	(9.00)
50–59	86.76	85.45	84.64	68.67	**75.10**	**95.58**	87.45	**74.47**	50.28	**50.56**
	(17.20)	(21.00)	(16.20)	(19.42)	(16.68)	(20.26)	(19.23)	(17.65)	(7.99)	(8.88)
60–69	84.44	83.25	83.81	67.06	74.57	93.71	85.09	74.38	49.23	50.20
	(18.03)	(2.99)	(16.29)	(19.23)	(16.83)	(20.73)	(2.74)	(17.87)	(8.29)	(9.42)
70–79	80.12	81.45	82.65	65.67	72.99	92.08	83.46	72.15	47.91	49.55
	(20.33)	(25.32)	(16.77)	(18.14)	(16.14)	(22.31)	(24.21)	(18.43)	(8.67)	(9.20)
80-	82.13	79.19	81.92	63.10	73.66	91.39	79.79	72.81	47.79	48.90
	(16.22)	(29.46)	(16.35)	(18.48)	(16.03)	(20.2)	(30.31)	(16.71)	(7.81)	(9.60)
F	17.571***	6.020***	4.481***	6.190***	1.469	2.874**	5.321***	1.484	19.245***	1.573
Education level										
Elementary school or lower	80.89	76.88	81.26	63.89	73.43	90.86	78.77	73.55	47.08	49.11
	(19.32)	(29.60)	(17.04)	(19.84)	(16.68)	(22.97)	(29.14)	(17.87)	(9.26)	(9.76)
Junior middle school	83.82	83.81	83.83	67.37	74.04	93.76	85.91	73.47	49.25	50.10
	(19.17)	(21.80)	(16.28)	(19.00)	(16.79)	(21.11)	(20.53)	(18.21)	(8.14)	(9.10)
Senior middle school	86.67	85.19	84.29	67.95	74.57	94.58	86.11	73.50	50.30	49.87
	(17.15)	(20.11)	(16.22)	(19.59)	(17.03)	(20.21)	(20.30)	(18.09)	(7.92)	(9.19)
College	**87.49**	**86.53**	**86.40**	69.76	**75.10**	**95.56**	**86.52**	**74.57**	**51.19**	50.10
	(16.97)	(16.52)	(15.43)	(18.93)	(16.87)	(20.27)	(18.62)	(17.55)	(7.50)	(8.930
University or higher	87.19	85.10	84.97	**70.22**	74.33	94.92	86.47	74.18	50.59	**50.28**
	(18.45)	(18.99)	(16.36)	(18.62)	(16.82)	(20.95)	(18.13)	(17.29)	(7.51)	(8.40)
F	15.515***	15.310***	8.168***	9.326***	0.989	3.837**	10.636***	1.897	21.921***	0.794
Marital status										
Not married yet	84.62	84.49	**85.98**	**68.56**	71.87	91.79	85.92	72.35	**50.24**	48.96
	(20.90)	(13.43)	(16.89)	(18.89)	(17.27)	(22.43)	(10.96)	(19.29)	(7.28)	(8.71)
Married currently	**86.08**	**84.90**	84.51	68.49	**74.76**	**94.85**	**86.00**	74.19	50.15	**50.16**
	(17.67)	(20.32)	(16.07)	(19.25)	(16.88)	(20.54)	(20.46)	(17.77)	(7.92)	(9.00)
Divorced or widowed	80.56	76.78	80.40	62.77	74.32	91.42	79.82	**74.30**	46.50	49.84
	(19.95)	(36.24)	(16.67)	(18.14)	(14.88)	(21.19)	(34.85)	(15.86)	(10.31)	(10.13)
F	11.015***	16.528***	9.777***	9.556***	8.058***	8.189***	9.744***	2.993	19.14***	5.571**
Ethnic group										
Han	85.68	84.51	**84.60**	68.31	74.56	94.49	85.75	74.10	50.01	50.08
	(18.27)	(20.55)	(16.13)	(19.14)	(16.77)	(20.73)	(20.42)	(17.78)	(7.98)	(8.95)
Hui	86.30	83.89	84.26	66.36	67.32	94.96	87.20	67.74	50.02	47.85
	(19.22)	(21.91)	(18.24)	(22.10)	(21.37)	(22.56)	(16.10)	(21.94)	(8.88)	(11.09)
Miao	70.56	**87.99**	89.11	67.99	68.89	88.89	89.91	61.78	49.07	47.31
	(31.37)	(6.81)	(14.46)	(11.86)	(15.77)	(22.05)	(6.29)	(25.85)	(6.61)	(8.98)
Wei	86.07	86.76	83.25	64.25	**82.50**	**100.0**	**96.43**	**78.50**	48.26	**55.38**
	(17.90)	(25.91)	(17.03)	(26.95)	(15.81)	(14.94)	(6.60)	(18.63)	(11.49)	(9.57)
Man	**86.71**	86.55	82.67	**70.24**	72.85	93.84	87.21	72.57	**50.50**	49.49
	(14.07)	(18.48)	(16.52)	(20.69)	(18.30)	(21.16)	(18.00)	(18.25)	(7.66)	(9.81)
Others	85.56	80.62	81.53	63.06	70.96	87.81	79.78	72.35	48.78	47.43
	(17.56)	(28.40)	(18.48)	(18.04)	(17.09)	(24.36)	(25.26)	(19.72)	(8.90)	(10.59)
F	1.344	0.915	1.085	1.629	3.030*	1.993	2.032	1.763	0.645	2.735*
Living environment										
Urban	85.63	84.47	84.41	68.11	74.47	94.21	85.73	73.98	49.95	50.01
	(18.28)	(20.84)	(16.23)	(19.25)	(16.83)	(20.87)	(20.58)	(17.93)	(8.00)	(9.05)
Suburban	85.54	84.79	**85.53**	69.48	**74.62**	95.49	85.19	**74.79**	50.32	**50.21**
	(17.62)	(16.59)	(15.64)	(18.02)	(16.80)	(20.72)	(17.77)	(17.37)	(7.65)	(8.40)
Rural	**88.17**	**85.59**	85.34	**70.84**	71.36	**96.75**	**87.04**	72.17	**51.30**	49.22
	(16.43)	(22.44)	(16.13)	(19.86)	(18.39)	(19.30)	(22.27)	(17.18)	(8.69)	(9.39)
F	1.223	0.215	0.923	1.991	2.133	1.458	0.375	1	1.798	0.721
Medical insurance										
Expenses of one’s own	84.56	86.05	**85.34**	**70.92**	74.00	94.17	86.16	73.51	50.55	49.88
	(21.11)	(17.78)	(15.90)	(20.51)	(17.79)	(21.04)	(19.14)	(19.77)	(7.86)	(9.26)
The new rural cooperative medical care	**88.43**	84.86	84.78	69.64	73.32	**95.59**	86.66	73.85	**50.84**	49.77
	(15.62)	(19.28)	(16.39)	(19.16)	(17.43)	(20.68)	(18.85)	(17.76)	(8.05)	(9.41)
Medical insurance for urban residents and workers	85.67	84.26	84.45	68.02	**74.49**	94.33	85.53	73.97	49.94	49.99
	(18.09)	(20.93)	(16.25)	(19.13)	(16.78)	(20.81)	(20.56)	(17.78)	(8.02)	(8.98)
Others	84.89	**88.32**	83.47	66.56	74.41	93.66	**89.93**	**76.01**	49.40	**51.30**
	(16.61)	(19.81)	(14.73)	(16.93)	(16.55)	(21.19)	(22.48)	(15.71)	(7.45)	(9.03)
F	2.16	2.506	0.563	3.404*	0.403	0.311	2.261	0.678	1.674	0.801
Chronic disease status										
Yes	**86.62**	**86.69**	**86.25**	**70.74**	**74.86**	**94.88**	**87.09**	**74.27**	**51.10**	**50.13**
	(19.00)	(15.92)	(15.43)	(19.28)	(17.16)	(20.80)	(17.26)	(18.43)	(7.32)	(8.82)
No	84.80	84.46	82.86	65.92	73.98	93.85	84.45	73.72	48.97	49.90
	(17.38)	(17.38)	(16.74)	(18.82)	(16.58)	(20.83)	(22.30)	(17.35)	(8.46)	(9.21)
T	−3.664***	−7.610***	−7.684***	−9.263***	−1.892	−1.799	−4.741***	−1.133	−8.719***	−0.785
Smoke										
Never	**85.79**	84.55	**84.59**	68.28	74.33	**94.42**	85.77	73.90	**50.07**	49.97
	(17.94)	(20.38)	(16.14)	(19.16)	(16.87)	(20.72)	(20.35)	(17.82)	(7.93)	(9.00)
Yes	84.96	**84.82**	84.16	**68.79**	**75.10**	94.28	**85.84**	**74.94**	49.76	**50.46**
	(20.35)	(20.61)	(16.28)	(19.24)	(16.93)	(21.27)	(20.01)	(18.61)	(8.06)	(9.19)
Give up	84.63	81.38	82.81	64.75	74.27	91.37	83.53	72.84	48.79	49.31
	(18.07)	(29.44)	(17.96)	(20.05)	(16.55)	(22.63)	(26.12)	(16.77)	(10.11)	(8.90)
F	0.723	1.395	0.823	2.137	0.530	0.941	0.684	1.092	1.338	1.167
Drink										
Yes	85.63	**84.68**	84.23	67.39	**75.43**	**94.71**	**86.05**	**75.05**	49.71	**50.57**
	(17.09)	(22.15)	(16.43)	(20.18)	(17.04)	(20.97)	(21.50)	(17.25)	(8.39)	(8.96)
No	**85.69**	84.49	**84.55**	**68.39**	74.26	94.30	85.68	73.83	**50.05**	49.93
	(18.36)	(20.41)	(16.16)	(19.04)	(16.84)	(20.80)	(20.29)	(17.97)	(7.94)	(9.03)
T	0.076	−2.33	0.473	1.271	−1.712	−0.488	−0.422	−1.690	0.894	−1.732
Having breakfast weekly										
Less than 2 days	85.70	84.58	84.69	68.32	74.41	94.42	**85.81**	74.01	50.06	50.03
	(18.30)	(20.58)	(16.07)	(19.19)	(16.89)	(20.89)	(20.35)	(17.87)	(7.95)	(9.00)
2-3days	85.45	83.70	82.89	67.19	74.83	94.46	85.35	74.62	49.32	50.35
	(17.24)	(21.87)	(16.53)	(19.06)	(16.29)	(19.12)	(21.39)	(17.63)	(8.36)	(8.77)
4-5days	85.45	83.70	82.89	67.19	74.83	94.46	85.35	74.62	49.32	50.35
	(17.24)	(21.87)	(16.53)	(19.06)	(16.90)	(19.12)	(21.39)	(17.63)	(8.36)	(8.77)
More than 5 days	**88.17**	**87.63**	**86.24**	**75.04**	**78.00**	**94.77**	85.44	**77.03**	**52.04**	**50.66**
	(14.21)	(17.79)	(15.98)	(20.04)	(17.77)	(21.78)	(22.81)	(17.87)	(7.42)	(10.03)
F	0.736	1.076	3.938**	4.362**	3.346*	1.185	0.045	4.087**	2.984*	2.797*
Sleep quality										
Very good	**86.00**	**86.51**	**86.27**	**72.09**	**75.72**	**94.88**	**86.94**	**74.91**	**51.05**	**50.46**
	(19.13)	(14.79)	(15.78)	(18.95)	(17.04)	(20.36)	(16.53)	(18.33)	(7.23)	(8.64)
Fairly good	85.83	84.67	84.54	68.01	74.37	94.50	85.97	74.05	50.01	50.07
	(18.04)	(20.61)	(16.08)	(19.03)	(16.85)	(20.84)	(20.38)	(17.76)	(7.99)	(8.99)
Fairy bad	82.59	76.11	79.11	60.83	71.18	90.85	78.90	70.37	46.87	47.94
	(19.70)	(31.72)	(17.99)	(19.61)	(16.15)	(22.06)	(28.95)	(18.19)	(9.37)	(10.37)
Very bad	81.52	79.51	75.09	57.69	69.20	88.58	76.63	69.79	46.26	46.93
	(16.82)	(26.24)	(15.82)	(20.89)	(16.60)	(16.82)	(29.27)	(14.39)	(8.49)	(8.02)
F	3.130**	17.979***	15.897***	27.419***	5.709**	3.345*	12.631***	4.824*	16.679***	5.403**

*PF=* physical functioning, *RP=* role limitations due to physical problems, *BP=* bodily pain, *GH=* general health, *VT=* vitality, *SF*= social functioning, *RE=* role limitations due to emotional problems, *MH*= mental health, *PCS=* physical component summary, *MCS=* mental component summary

The highest average scores in each dimension were marked bold word

*P-value < 0.05. **P-value < 0.01. ***P-value < 0.001

**Table 4:** Socio-demographic characteristics and lifestyle behaviors factors associated with scale scores of SF-36 resulted from multiple linear regression

Scales	PF	RP	BP	GH	VT	SF	RE	MH	PCS	MCS
Gender	0.030*	0.031*		0.032*					−0.044**	
Age	−0.126***	−0.071***	−0.061***	−0.071***		−0.035*	−0.44**		−0.137***	
Educational level										
Senior high school				−0.052**				−0.041*		
Junior middle school	−0.085***			−0.069***				−0.044*	−0.90***	
Primary school or lower	−0.084***	−0.096***	−0.055***	−0.080***		−0.047**	−0.091***		−0.105***	
Marital status										
Currently married			−0.033*		0.062***	0.053**		0.038*		0.052**
Divorced or widowed	−0.045**	−0.075***	−0.069***	−0.060***			−0.06***		−0.083***	
Ethnic groupa										
Hui					−0.038**					
Miao	−0.034*							−0.028*		
Living environment										
rural					−0.028*					
Medical insurance										
The new rural cooperative medical care	0.040*									
Medical insurance for urban residents and workers		−0.40*		−0.031*						
Chronic diseases	0.050***	0.102***	0.104***	0.126***			0.065***		0.118***	
Having Breakfast frequency weekly										
4–5 days				−0.105**					−0.077*	
2–3 days				−0.104**					−0.084*	
Less than 2 days			−0.049*	−0.073**	−0.064**			−0.065**	−0.057**	−0.046*
Sleep quality										
Still good			0.252**	0.274***	0.184*				0.212**	
Not good			0.239**	0.220*	0.187*				0.191*	

*Variable coding.* Gender (male = 1, female = 0) and chronic disease (yes = 1, no = 0) were the binomial variables. Educational level was assessed with a set of four dummy variables (college = 1, other = 0; senior high school = 1, other = 0; junior middle school = 0, other = 1 and primary school or lower = 1, other = 0) with university or higher as the reference category; marital status was assessed with a set of two dummy variables(currently married = 1 and divorced or widowed = 0), with currently unmarried as the reference category; ethnic group was assessed with a set of five dummy variables (Hui = 1, other = 0; Miao = 1, other = 0; Wei = 1, other = 0; Man = 1, other = 0 and Zhuang = 1, other = 0) with Han as the reference category; medical insurance assessed a set of tw0 dummy variables (the new rural cooperative medical care = 1, medical insurance for urban residents and workers = 0) with expenses of one’s own as the reference category; having breakfast weekly assessed with a set of three dummy variables (less than 2 days = 1, other = 0; 2–3 days = 1, other = 0 and 4–5 days = 1, other = 0) with more than 5 days as the reference category; sleep quality also assessed with a set of three dummy variables (fairly good = 1, other = 0; not good = 1, other = 0 and very poor = 1, other = 0) with very good as the reference category

*PF* = physical functioning, *RP* = role limitations due to physical problems, *BP* = bodily pain, *GH* = general health, *VT* = vitality, *SF* = social functioning, *RE=* role limitations due to emotional problems, *MH=* mental health, *PCS=* physical component summary, *MCS=* mental component summary

*P-value < 0.05. **P-value < 0.01. ***P-value < 0.001

Lifestyle behaviors for the risk factors of chronic diseases described in details relating to HRQoL were calculated as well. Non-smoking residents had better physical health than the ones who were smoking, so are the non-drinkers. Residents who smoke or drink had a better mental health especially in RP, VT, RE and MH dimensions. Scores of each dimension of SF-36 increased when the sleep quality was in a higher level. Residents having breakfast more than 5 days weekly have higher scores.

Significant differences (p value below 0.05) were found in each dimension of SF-36, lifestyle behaviors and socio-demographic characteristics (Table 3): (1) sleep quality was found significantly different in each dimension scores of SF-36, while living environment and drinking and smoking habits were not; (2) gender, age, educational level and chronic disease status were found significantly different in all physical health dimensions, so as in PCS scores; (3) frequency of having breakfast weekly was found significantly different except in PF and RP dimensions; (4) In all physical health dimensions and RE dimension, significantly different were assessed in chronic disease status; (5) medical insurance was found significantly different only in GH dimension, however, marital status was found significantly different in all dimensions except MH; (6) ethnic groups and frequency of having breakfast weekly were significantly different in VT dimension and mental health summary scores (MCS). Furthermore, only breakfast frequency weekly was found significantly different in mental health (MH) dimension; (7) age and educational level were found significantly different in SF and RE (besides chronic disease status) dimensions.

### Multiple linear regression analysis of factors related to HRQoL

To have access to find the factors associated with the residents’ HRQoL, socio-demographic characteristics and lifestyle behaviors were used as the independent variables, while SF-36 scores in eight dimensions dependent variables. Multiple linear stepwise regressions results were evaluated to the factors affecting HRQoL (Table 4): For socio-demographic characteristics: (1) gender was positively associated with PF, RP and GH dimensions, while was negatively associated with PCS; (2) age was negatively associated with all dimensions except VT, MH and MCS; (3) educational level in senior high school was negatively associated with GH and MH; junior middle school was negatively associated with PF, GH, MH and PCS; primary school or lower educational level was negatively associated with all dimensions except VT, MH and MCS; (4) currently married was positively associated with VT, SF, MH and PCS dimensions while negatively with BP; divorced or widowed was associated negatively with all physical dimensions and RE; (5) chronic disease was positively associated with all dimensions of physical health and RE dimension. Living environment was almost not associated with SF-36 except VT dimension in rural. Lifestyle behaviors such as frequency of having breakfast weekly in lifestyle behaviors was the factors negatively associated with SF-36 in GH and PCS dimensions. Sleeping still good and not good were positively associated with BP, GH, VT and PCS dimensions. Nevertheless, drinking and smoking habits were not associated with HRQoL scores.

## Discussion

This study investigated a large population-based sample of HRQoL in northeast China. The residents’ physical health was better than their mental health, which was in accordance with the earlier study in other areas of China [2]. According to the above results, age, educational level, marital status, quality of sleep and chronic disease status were the factors related to HRQoL scores. Age was one of the most common and important factor associated with HRQoL in previous researches [2, 3, 10, 22]. In our study, younger community residents had higher scores in physical health, while middle-age group had better mental health. Earlier study found that the rural middle-age residents’ MCS scores were higher than PCS scores [27], which was similar to the results among urban areas in previous studies [2, 28–30]. Increasing age might lead to deteriorating physical and mental health. Therefore, younger residents had better physical health. Educational level might directly or indirectly associate with health-related quality of life in previous surveys [16]. Education means more opportunities to find a job with a better salary and better living conditions in both urban and rural areas [17]. Furthermore, it is more convenient for highly educated residents to obtain information of health in a more efficient ways so as to adjust their health conditions in time. Consequently, residents with higher education would obtain higher HRQoL might be due to their higher social class and economic status [16]. Marital status was the factor partly associated with the physical and mental health dimensions in HRQoL. Family and social support of unmarried and widowed or divorced patients were less than that of the married patients [31]. Mutual support and help between couples may positively influence the mental health. But relationships between couples needed to be taken care of, such as their children and parents, may affect their physical health. 4-2-1 family mode was extremely increasing in Chinese families, which means a child should support their parents who also need to support their parents together. While current unmarried people have fewer burdens in their families, therefore their physical health seems be better than those married. Sleep quality strongly influenced the residents’ physical and mental health. Residents who had better quality of sleep had higher scores of HRQoL. Sleep problems resulting from ageing were the factors associated with sleep quality [32] since younger adults have longer sleep than middle-aged and older adults. Chronic conditions were a positively factor related to HRQoL. Low quality of sleep may affect immune system, the ability to function and social activities, and may have an impact on physical and mental health in HRQoL [33]. The impact of chronic conditions in general populations was reflected more in PCS than in MCS scores [15]. Compared to the rural areas, adequate health information and knowledge, higher level of basic infrastructure, higher incomes, availability of health services may help urban residents prevent chronic diseases in northeast China [34]. The convenience might also be helpful for patients suffering from chronic diseases to hold an optimistic attitude to cure the disease. Higher consciousness of health and level of medical treatment were available to improve their physical and mental health.

The smoking and drinking habits are the major factors causing the decline of physical condition [35, 36]. A review indicated that unhealthy lifestyle behaviors including smoking and drinking were independent predictors of chronic conditions [37]. In this study, chronic disease status was a significant factor of SF-36 scores, whereas smoking and drinking behaviors did not affect HRQoL. In previous studies, smoking was almost hardly associated with the SF-36 scores [38]. The percentage of smokers in our study was 10.4 %, lower than the Chinese smoking rate in 2014 (28.1 %) [39]. It may be considered that most of the smokers did not present subjective symptoms over a threshold [40]. Moderate drinking may have a protective effect on the cardiovascular system [41, 42]; however, alcohol consumption may change because of health conditions [43]. Unlike a study of civil servant in China [44], drinking was not observed as the major association with HRQoL. The reason might be the higher rate of civil servants (62.52 %) than the community residents (13 %).

Interestingly, living environment was not the factor of HRQoL. Residents living in urban had lower physical and mental health than other living regions. The environment might be defined as the sum of all the conditions and elements, which make up the surroundings and influence the development and well-being of the individual [45]. It is emphasized on dwelling conditions in previous studies [18, 46 and 47]. The satisfactory of dwelling conditions reported better health and higher quality of life [18]. The satisfactory dwelling conditions residents considered might be unpolluted environment, which was not concerned until the panic like haze appears. Although a transition of industrial structure has been ongoing in northeast of China, the traditional consciousness of relationship between health and environment might not change. Therefore, transformations of the health concept of community residents would be strengthened to focus on the relationship between environment changes and health issues.

In spite of the characteristics in northeast of China, the reliability in SF was low in this study, which was consistent with the survey of Shanghai. Conceptualization of social function might mislead residents to understand [2]. Furthermore, reliability of VT was lower than SF, which was much lower than other studies [3, 5, 10]. Chinese people have the certain character that they are hardly express their real feelings under most circumstances, even hardly in a direct way to their families [2]. However, characteristic of people in northeast is a contradiction case. Due to multiple history, climate and nature environment factors, the characteristics of cultural personality in northeast of China was different from other areas of China, which were brave, frank, warm and bold. The expression way in northeast of China is more directly than other areas especially in the south area [47]. It makes them easier to participate in social activities more energetically, which might be helpful to their health. Nevertheless, though most factories have moved from the center of the city, decades of heavy industry in Shenyang had a negative impact on residents’ health. In addition, northeast people prefer to drink and smoke in ordinary life for geographical environment and history cultural practice [48], which created bad lifestyle habits influencing health. Laziness psychological feelings dependent on the weather and poor management make northeast people lack the innovation, which might influence their vitality dimension in health-related quality of life.

There are a number of limitations considered in this study. First, the study design was a cross-sectional research, therefore conclusions on the causality of the associations between residents and HRQoL could not be derived. A longitudinal study is needed in future studies. In addition, there are other potential factors which are not taken into account in this study, such as the district-level variations among the residential areas, and the provider-level disparities in the quality of the care provided by different community health centers, as the organizational model of primary care itself may has an impact on primary care quality and health utilization [49, 50]. Although residents were selected almost all from the urban area where community health centers were randomly selected, some underdeveloped regions did not incorporate into the survey area. The representativeness of sample might have potential selection bias. Moreover, the participants who were living in the same household were not taken into account in the survey. It might impact on the results since the similar lifestyle behaviors. Finally, interviewers might mislead the results when the residents received their explanation of the questions. Despite of these limitations above, the sample size was large in this study. Furthermore, the analysis results could provide prospective conclusions relating to HRQoL and its factors in Northeast China.

## Conclusions

In conclusions, we assessed the relationships between HRQoL and affected factors in a large-scale population in Shenyang, Northeast China. The results of HRQoL indicated their particular characters through comparing with other areas of China. Furthermore, factors influencing HRQoL were evaluated, including age, educational level, marital status, ethnic group, and chronic disease status and sleep quality. However, smoking and drinking habits were not the factors.

According to these results, policies of public health in order to highlight the long-term supervision and treatment of community residents’ chronic diseases might improve the health-related quality of life on the basis of the community health management. Community residents’ health management includes regular check-ups and special education like eating habits, chronic disease prevention and control etc. Furthermore, a good way of life especially eating habits could be advocated in order to improve residents’ health-related quality of life.

### Availability of data and materials

Not applicable.

## Abbreviations

HRQoL: Health-related quality of life
PF: Physical functioning
RP: Role limitations due to physical problems
BP: Bodily pain
GH: General health
VT: Vitality
SF: Social functioning
RE: Role limitations due to emotional problems
MH: Mental health
PCS: Physical component summary
MCS: Mental component summary
